# Significant social events and increasing use of life-sustaining treatment: trend analysis using extracorporeal membrane oxygenation as an example

**DOI:** 10.1186/1472-6939-15-21

**Published:** 2014-03-04

**Authors:** Yen-Yuan Chen, Likwang Chen, Tien-Shang Huang, Wen-Je Ko, Tzong-Shinn Chu, Yen-Hsuan Ni, Shan-Chwen Chang

**Affiliations:** 1 Department of Social Medicine, National Taiwan University College of Medicine, Taipei, Taiwan; 2 Department of Medical Education, National Taiwan University Hospital, Taipei, Taiwan; 3 Institute of Population Health Sciences, National Health Research Institutes, Miaoli, Taiwan; 4 Department of Internal Medicine, National Taiwan University Hospital, Taipei, Taiwan; 5 Department of Surgery, National Taiwan University College of Medicine, Taipei, Taiwan; 6 Department of Traumatology, National Taiwan University Hospital, Taipei, Taiwan; 7 Department of Primary Care Medicine, National Taiwan University College of Medicine, Taipei, Taiwan; 8 Department of Pediatrics, National Taiwan University College of Medicine, No. 1, Road Ren-Ai sec. 1, Taipei 100, Taiwan; 9 Department of Medical Genetics, National Taiwan University Hospital, Taipei, Taiwan; 10 Department of Internal Medicine, National Taiwan University College of Medicine, Taipei, Taiwan

**Keywords:** Life-sustaining treatment, Extra-corporeal membrane oxygenation, Cardiopulmonary resuscitation, Media, Trend

## Abstract

**Background:**

Most studies have examined the outcomes of patients supported by extracorporeal membrane oxygenation as a life-sustaining treatment. It is unclear whether significant social events are associated with the use of life-sustaining treatment. This study aimed to compare the trend of extracorporeal membrane oxygenation use in Taiwan with that in the world, and to examine the influence of significant social events on the trend of extracorporeal membrane oxygenation use in Taiwan.

**Methods:**

Taiwan’s extracorporeal membrane oxygenation uses from 2000 to 2009 were collected from National Health Insurance Research Dataset. The number of the worldwide extracorporeal membrane oxygenation cases was mainly estimated using Extracorporeal Life Support Registry Report International Summary July 2012. The trend of Taiwan’s crude annual incidence rate of extracorporeal membrane oxygenation use was compared with that of the rest of the world. Each trend of extracorporeal membrane oxygenation use was examined using joinpoint regression.

**Results:**

The measurement was the crude annual incidence rate of extracorporeal membrane oxygenation use. Each of the Taiwan’s crude annual incidence rates was much higher than the worldwide one in the same year. Both the trends of Taiwan’s and worldwide crude annual incidence rates have significantly increased since 2000. Joinpoint regression selected the model of the Taiwan’s trend with one joinpoint in 2006 as the best-fitted model, implying that the significant social events in 2006 were significantly associated with the trend change of extracorporeal membrane oxygenation use following 2006. In addition, significantly social events highlighted by the media are more likely to be associated with the increase of extracorporeal membrane oxygenation use than being fully covered by National Health Insurance.

**Conclusions:**

Significant social events, such as a well-known person’s successful extracorporeal membrane oxygenation use highlighted by the mass media, are associated with the use of life-sustaining treatment such as extracorporeal membrane oxygenation.

## Background

Extracorporeal membrane oxygenation (ECMO), a critical life-sustaining treatment, works as a modified heart-lung machine, sustaining severely ill patients for several days or weeks while the patients await treatment for or undergo recovery from cardiac or respiratory failure. In 1972, Hill et al. reported the survival of a severely traumatized 24-year-old patient who presented acute respiratory distress syndrome and was supported using ECMO during the acute phase of the disease [1]. To date, ECMO has been used to sustain the lives of patients who exhibit respiratory or cardiac failure refractory to conventional intensive treatments [2-4].

As presented in the report “Extracorporeal Life Support Registry Report International Summary July 2012,” the annual number of ECMO patients gradually increased from 1,644 in 1990 to 3,346 in 2011. Since 1990, a total of 50,667 patients have received ECMO for cardiac or respiratory failures, or ECMO-assisted cardiopulmonary resuscitation [5].

In Taiwan, ECMO was first administered at the end of the 1990s, but such treatment was not fully covered by National Health Insurance (NHI) until December 1, 2002. ECMO was involved in two high-profile cases in Taiwan in 2006 and 2007; the spouse of a mayor who was a famous movie star [6,7] and a TV celebrity [8]. These cases attracted substantial attention, demonstrating that administering ECMO can save severely ill and dying patients. In 2008, a press conference held by a university-affiliated medical center in Northern Taiwan further highlighted the use of ECMO to assist patients receiving cardiopulmonary resuscitation [9,10].

Previous studies have focused on the outcomes of ECMO use in North America and Europe [11-14] and Taiwan [15,16]. Identifying ECMO use trends is crucial for examining how the use of life-sustaining treatments, such as ECMO, relates to significant social events headlined by the media. Therefore, in this study, secondary data from the Taiwan National Health Insurance Research Database (NHIRD) were used to estimate trends in the crude annual incidence rate (CAIR) for ECMO use in Taiwan between January 1, 2000, and December 31, 2009. The objectives of this study were the following: (1) to compare the trend of ECMO use in Taiwan with that worldwide; and (2) to examine the influence of significant social events on the trend of ECMO use in Taiwan.

## Methods

### Data collection

We used SAS 9.3 software (Statistical Analysis System 9.3 for Windows PC) to construct our analytical data file. We used secondary data from the Taiwan NHIRD, which was provided by the Bureau of NHI and managed by the National Health Research Institutes. This large database comprises all NHI claims and registration data and is freely available for research purposes. By using the Taiwan NHIRD, we extracted and organized the registration and hospital care data for all patients who were supported using ECMO between January 1, 2000, and December 31, 2009. We also estimated the annual population of Taiwan on July 1 of each year from 2000 to 2009 by using the Taiwan NHIRD.

The annual number of worldwide ECMO cases during the study period was obtained from the annual number of ECMO cases reported in Extracorporeal Life Support Registry Report International Summary July 2012, excluding the annual number of ECMO cases in Taiwan. We obtained the world population estimate for each year starting from 2000 from the “World Population Prospects: the 2010 Revision,” published by the Department of Economic and Social Affairs, United Nations Population Division [17]. We considered the Least Developed Countries incapable of initiating ECMO use and omitted these countries from the denominator of the worldwide CAIR [18]. Thus, the world population estimates for each year was equal to the total world population minus the population of the Least Developed Countries and Taiwan.

We defined the CAIR as the number of ECMO cases per million people in a single year. We derived the CAIR for Taiwan and the world for each year, comparing the CAIR of ECMO use in Taiwan with that in the rest of the world.

### Statistical analysis

We plotted two trends: (1) the CAIR of ECMO use in Taiwan; and (2) the CAIR of worldwide ECMO use. We conducted linear regression of the CAIR of ECMO use in Taiwan on Year (explanatory variable), and the CAIR of the worldwide ECMO use on Year (explanatory variable). We recognized the increasing trend of each model if the coefficient of explanatory variable in the model is a positive value with statistical significance. The goodness-of-fit of each regression model was examined using the value of the adjusted R square. To ascertain whether the CAIR of ECMO use in a given year was autocorrelated, we derived the Durbin-Watson statistic for each linear regression model. We used the Pearson correlation coefficient to examine the linear correlation between Taiwan’s CAIR and the worldwide CAIR in a year.

To examine whether significant social events affected trends in the use of life-sustaining treatments, we performed joinpoint regression analysis for the trend of ECMO use in Taiwan and the trend of ECMO use in the rest of the world. Joinpoint linear regression is a statistical method used to determine the number of segments needed to adequately account for the linear relationship between two variables. The permutation test is used repeatedly in Joinpoint Regression Program to select the final joinpoint linear regression model. What the program does is to try to choose the smallest number of joinpoints such that if one more joinpoint is added, the change of the model is not statistically significant [19,20]. We supplied the minimal and the maximal number of joinpoints as zero and two respectively, and the minimal number of data point between two joinpoints as two.

All statistical analyses in this study, except joinpoint regression, were carried out using the software package of STATA 11.0/MP for Windows PC. We performed joinpoint regression using Joinpoint Regression Program 3.5.2 for Windows PC which was developed by National Cancer Institute in the United States of America [21]. This study was approved by Research Ethics Committee in National Taiwan University Hospital (No.201212043W).

## Results

The annual number of ECMO cases in Taiwan nearly quadrupled during the last decade, from 257 cases in 2000, to 886 cases in 2009. By comparison, the annual number of worldwide ECMO cases has increased by 37.63%, from 1,600 cases in 2000 to 2,202 cases in 2009. The CAIR of ECMO use in Taiwan has dramatically increased, from 11.71 cases per million people in 2000 to 37.47 cases per million people in 2009. By comparison, the CAIR of worldwide ECMO use increased from 0.29 cases per million people in 2000 to 0.37 cases per million people in 2009. The CAIR of ECMO use in Taiwan was more than 40-times that the CAIR of worldwide ECMO use. Table 1 shows the disparities in ECMO cases and the CAIR of ECMO use in Taiwan and throughout the world.

**Table 1:** The comparison of extracorporeal membrane oxygenation use between the world and Taiwan

	**Case**		**Incidence**	
	**Taiwan**	**World**	**Taiwan**	**World**
**Year**	**N**	**N**	**CAIR**^**a**^	**CAIR**
2000	257	1600	11.71	0.29
2001	274	1579	12.25	0.29
2002	311	1594	13.81	0.29
2003	461	1504	20.37	0.27
2004	435	1472	19.05	0.26
2005	393	1778	16.98	0.31
2006	413	1915	17.73	0.33
2007	666	1872	28.39	0.32
2008	857	1857	36.28	0.31
2009	886	2202	37.47	0.37

^a^CAIR denotes for “crude annual incidence rate,” which means the annual number of ECMO cases per million people registered in National Health Insurance in the year.

1. “Case” in World is equal to the total cases in the world minus the total cases in Taiwan.

2. The estimate of Taiwan population for each year was obtained by using the total number of people registered in National Health Insurance on July 1 in the year.

3. The estimate of World population for each year in this study was equal to the total number of the world population minus the total population in the least developed countries as shown in “World Population Prospects: the 2010 Revision”, and also minus the total of Taiwan population.

From 2000 to 2003, the CAIR of ECMO use in Taiwan increased, and gradually decreased from 2003 to 2005. However, the CAIR of ECMO use in Taiwan has shown a dramatic upward trend since 2006. Linear regression analysis was used to examine the CAIR of ECMO use in Taiwan, indicating that the CAIR of ECMO use has significantly increased over time (β coefficient = 2.8058, 95% C.I. = 1.6925 ~ 3.9192, *p* < .01), attaining an adjusted R square value of 0.7846. By comparison, the worldwide CAIR of ECMO use slightly decreased beginning in 2000, reaching its lowest level in 2004, after which the worldwide CAIR gradually increased to its highest level in 2009. The upward trend of the worldwide CAIR of ECMO use was statistically significant (β coefficient = 0.0075, 95% C.I. = 0.0016 ~ 0.0135, *p* = .02), attaining an adjusted R square value of 0.4548. However, comparing the CAIR of ECMO use in Taiwan with the worldwide CAIR for each year showed no significant linear correlation (r^2^ = 0.6116, *p* = .06). Joinpoint regression for the trend of Taiwan’s CAIRs finally selected the model with one joinpoint in 2006 as the best-fitted model. By comparison, the best-fitted model for the worldwide CAIR selected by joinpoint regression was the model with no joinpoint.

We used the Durbin-Watson test to examine whether any two consecutive CAIRs showed positive or negative autocorrelations in the two trends. For the model that had one regressor (k = 1) and 10 CAIRs (n = 10), the lower and upper limits of the 5% significance values were 0.879 (*d*_*L*_) and 1.320 (*d*_*U*_), respectively. Neither the Taiwanese nor worldwide CAIR of ECMO use showed positive or negative autocorrelations.

## Discussion

### Main findings

Although the outcomes of ECMO have been extensively researched [14,22-24], no study has investigated the trend of ECMO use or how significant social events influence such trends. We showed that ECMO use in Taiwan substantially increased over time with a joinpoint in 2006, implying a particularly high demand for ECMO thereafter. The trend of worldwide ECMO use also increased over time, but no joinpoint was present between 2000 and 2009, indicating that the trend did not change during this period. We did not identify any positive or negative autocorrelations in the trends of ECMO use, suggesting that significant social events in 2006 were associated with the dramatic change in the trend of ECMO use in Taiwan.

### Dissimilarities between Taiwan and the world in ECMO use

Our results showed that Taiwan has a higher demand for ECMO as a life-sustaining treatment compared with the rest of the world as indicated by the follows: first, the CAIR of ECMO use in Taiwan was approximately 40-times higher compared with that rest of the world; second, the yearly increment of CAIR in Taiwan was 2.8058 cases per million people, which was substantially higher compared with the yearly increment of worldwide CAIR, 0.0075 cases per million people.

Several possibilities may account for the high demand for ECMO as a life-sustaining treatment in Taiwan: first, the higher incidence of cardiac or respiratory failure refractory to conventional treatments in Taiwan than that of the rest of the world; second, more relaxed indications for initiating ECMO in Taiwan than the indications for initiating ECMO in the rest of the world [2-4,25]. The first possibility is not supported because no evidence shows that the incidence of cardiac or respiratory failure refractory to conventional treatments is higher in Taiwan compared with the rest of the world. The second possibility is also not supported because no study has examined whether the indications for initiating ECMO in Taiwan and the rest of the world are strictly followed. However, it may be likely that the indications for initiating ECMO in Taiwan are more relaxed than those in the rest of the world due to various reasons. This needs more studies to examine whether the indications are more relaxed, as well as the reasons associated with why the indications are more relaxed.

Health-related information demonstrated on the media may influence the general population’s provision and health-related behaviors, such as patients/family members’ consideration of requesting ECMO [26] and physicians’ ethical consideration of initiating this life-sustaining treatment. As indicated by cases of another life-sustaining treatment, cardiopulmonary resuscitation, the media often presents audiences with overtly optimistic information regarding the survival rates of patients who receive cardiopulmonary resuscitation. Such reports may cause the general population to mistakenly believe that cardiopulmonary resuscitation always saves patients; thus people may be inclined to request cardiopulmonary resuscitation when family members experience cardiac or respiratory arrest [27]. Therefore, patients and their family members in Taiwan may mistakenly believe that ECMO is always successful at saving critically ill patients, and becoming inclined to request ECMO as a life-sustaining treatment for the critically ill patients. This could explain why the demand for ECMO is considerably higher in Taiwan compared with the rest of the world.

We located no joinpoint for worldwide CAIR trend; however, the trend with a joinpoint in 2006 was the best-fitted model for the CAIR of Taiwan. The Durbin-Watson test showed no positive or negative autocorrelations between any two consecutive CAIRs for the ECMO use trend in Taiwan (Table 2), indicating that the abruptly increase in ECMO use after 2006 cannot be simply explained by the CAIRs before 2006. There may be some other issues associated with the trend change, such as famous personnel’s successful ECMO use headlined by the media.

**Table 2:** Linear regression analyses for the trends of extracorporeal membrane oxygenation use over time

	**Coefficients**^**a **^**for incidence rate**		**Durbin-Watson tests for autocorrelation**^**d**^		**Joinpoint regression analysis**
	**β (95% CI**^**c**^**)**	***p *** **value**	**Durbin-Watson Statistic**	**Status**	**Joinpoint**
Taiwan’s CAIR^b^	2.8058 (1.6925–3.9192)	<0.001	1.014	Indecision	One joinpoint at 2006
The World’s CAIR	0.0075 (0.0016–0.0135)	0.019	1.485	No autocorrelation	No joinpoint

^a^“Coefficients” indicates changes in incidence rates per million people when the time changed by one year.

^b^CAIR denotes for “crude annual incidence rate,” which means the annual of ECMO cases per million persons registered in National Health Insurance in the year.

^c^CI denotes for “confidence interval”.

^d^For 5% significance points, levels below 0.879 indicate significant positive autocorrelation; levels between 0.879 and 1.320 indicate zone of indecision; levels between 1.320 and 2.680 indicate no autocorrelation; levels between 2.680 and 3.121 indicate zone of indecision; levels above 3.121 indicate significant negative autocorrelation.

### Significant social events and extracorporeal membrane oxygenation use in Taiwan

**Figure 1:**
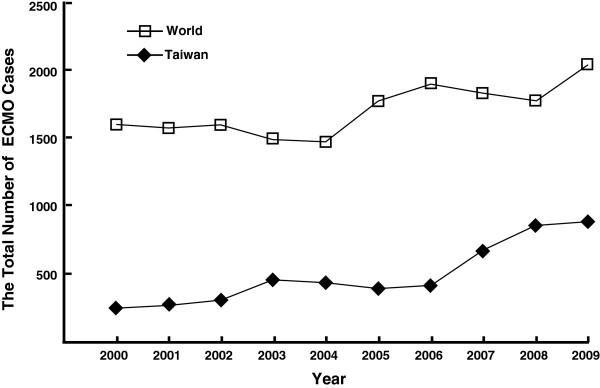
The comparison of Taiwan’s extracorporeal membrane oxygenation cases with the worldwide extracorporeal membrane oxygenation cases.

**Figure 2:**
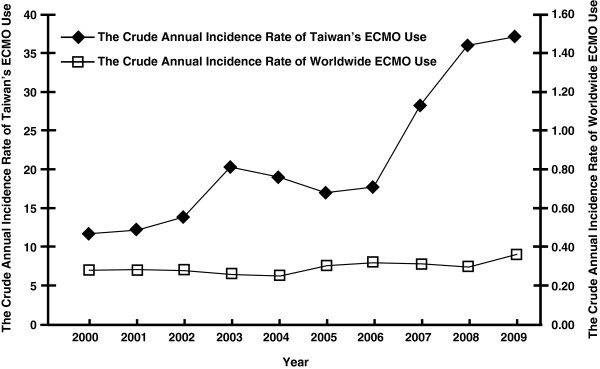
The comparison of Taiwan’s crude annual incidence rates of extracorporeal membrane oxygenation use with the worldwide crude annual incidence rates of extracorporeal membrane oxygenation use.

ECMO use has been fully covered by NHI in Taiwan since December 1, 2002. As expected, ECMO use showed an upward trend following this policy change (Table 1, Figures 1 and 2). The number of ECMO cases in Taiwan increased from 311 in 2002 to 461 in 2003, and the CAIR of ECMO use increased from 13.81 in 2002 to 20.37 in 2003. Nevertheless, no joinpoint was identified in 2002 by employing joinpoint regression (Table 2). Notably, ECMO use in Taiwan slightly decreased from 2003 to 2005. Thus, the full coverage of ECMO by NHI was associated with the increasing initiation of ECMO use in Taiwan in 2003, but not between 2003 and 2005. In addition, we only examined the trend of ECMO use in Taiwan from 2000 to 2009. H1N1 pandemic in 2009, which may be associated with more ECMO use in the rest of the world, absolutely did not have any influence on the trend change during the study period in Taiwan [28,29].

From the end of 2006 to the beginning of 2007, a TV celebrity and the spouse of a mayor recovered following the long-term use of ECMO to sustain their lives. The media highlighted their stories of being successfully rescued by ECMO. Health-related information presented by the media, particularly stories featuring celebrities and other notable people, may influence the general population’s medical decision-making of requesting life-sustaining treatments such as ECMO [26].

Our study showed that the survival of the TV celebrity and the mayor’s spouse after the long-term ECMO use may have led the general population to mistakenly believe that ECMO always saves patients from all life-threatening conditions. Therefore, the family members of severely ill patients in Taiwan may be more likely to request ECMO, and the attending physicians, usually in fear of law suits [30,31], are more likely to perform ECMO without carefully deliberating its suitability. As a result, an abruptly upward trend of ECMO use occurred following 2006, immediately after the significant social events highlighted in newspapers and TV programs. In addition, our study also showed that the full coverage of ECMO use by NHI may not significantly influence physicians’ judgment for initiating ECMO or the patients/family members’ preferences for requesting ECMO. The medical information of celebrities or other notable persons had a greater influence on the trend of ECMO use in Taiwan than the policy to fully cover ECMO use by NHI.

Medical decision-making to request, withhold or withdraw life-sustaining treatment is a critical issue in bioethics. Physicians assist in medical decision-making by providing medical information and suggestions based on scientific and humanistic principles, and do so with respect for patient autonomy. By comparison, patients usually make their medical decisions based on not only the medical information and suggestions given by the physicians, but also their personal values, preferences, and past medical experiences [32]. Consequently, significant social events highlighted by the media may influence patients’ and family members’ values and preferences, thus influencing their medical decision-making to withhold, withdraw or request life-sustaining treatments such as ECMO.

One of the social events, the mayor’s spouse who was a famous movie star, not only highlighted on newspapers and internet web pages, but also extensively reported by TV news programs [33]. Several years after the event, a local TV news program showed the lasting influence of the significant social event on using ECMO. A woman who stopped at a traffic light was struck by a falling object and severely injured. Cardiopulmonary resuscitation was immediately performed, after which, the victim was transported to a nearby hospital in deep coma (Glasgow Coma Scale = 4). Her husband broke down while being interviewed by the TV reporter, and said “I hope the health care team tries as hard to resuscitate my wife as the health care team members did for the mayor’s spouse” [34]. This case suggests that social events extensively reported by the media can influence patient and family member decisions to request life-sustaining treatments such as ECMO.

We do not consider our examples of the significant social events and the dramatic upward trend of ECMO use in Taiwan following 2006 to be causally related; a causal relationship must meet stringent requirements such as a temporal relationship, strength of association, a dose–response relationship, and so on [35]. However, our results satisfied the following two criteria for judging whether a relationship is causal: first, the trend of ECMO use in Taiwan immediately increased following the two significant social events, indicating a temporal relationship between the events and the increase in ECMO use; second, we measured the strength of the relationship by estimating relative risk levels and determined that the risk of ECMO use in 2007 was 1.6016-times more than the risk of ECMO use in 2006 (*p* < .01), indicating a strong association. Thus, the relationship between the aforementioned social events and the dramatic upswing in ECMO use in Taiwan following 2006 is more than a mere statistical relationship.

### Strengths and limitations

The media strongly influences public understanding of medical science, social controversies, and ethical considerations. In addition to examining the trends of ECMO use in Taiwan and throughout the world, we investigated how specific and noteworthy social events influenced the demand for ECMO use. By using data from the Taiwan NHIRD, we showed that significant social events highlighted by the media, such as a celebrity being saved using ECMO, are associated with the increasing demand for ECMO as a life-sustaining treatment.

Our study has the following limitations. First, concerns may exist regarding the reliability of ECMO use in Taiwan and throughout the world. We collected data regarding ECMO cases in Taiwan from the NHIRD, which contains all NHI claims and registration data. Although NHI covers 99.91% of the population in Taiwan [36], health care professionals could have provided ECMO to a small number of uninsured patients. In addition, we obtained the worldwide ECMO use data from Extracorporeal Life Support Registry Report International Summary July 2012, which was reported by the Extracorporeal Life Support Organization. The number of worldwide ECMO cases may be underestimated because not all medical centers report ECMO cases to the Extracorporeal Life Support Organization [37]. Furthermore, the denominator for calculating the worldwide CAIR was the population of the world minus the population in the Least Developed Countries. Therefore, the comparisons of CAIRs between Taiwan and the rest of the world may be of concerns. Nevertheless, the higher demand for ECMO in Taiwan compared with the rest of the world is convincing, and the significant social events are associated with the increasing ECMO use in Taiwan with statistical significance. Second, we did not ascertain whether the ECMO cases in Taiwan and in the rest of the world strictly followed the indications for initiating ECMO, namely, severe cardiac or respiratory failure refractory to conventional treatments.

## Conclusions

ECMO use is increasing more rapidly in Taiwan compared with the rest of the world. In this study, we examined the current practice of ECMO by using the largest dataset in Taiwan. Our findings showed that full coverage of ECMO by NHI did not substantially encourage ECMO use. By contrast, significant social events highlighted by the media, such as the successful use of ECMO to treat celebrities, were strongly associated with the increasing use of ECMO. Our results suggest that additional studies are required to determine the characteristics of the significant social events to predict the occurrence of trend change, how the information presented by the media affects medical decision-making, the trends of ECMO use based on age and sex, and the social and ethical implications of the outcome and disparities of ECMO use in Taiwan.

## Abbreviations

ECMO: Extracorporeal membrane oxygenation; NHI: National Health Insurance; NHIRD: National Health Insurance Research Database; CAIR: Crude annual incidence rate.

## Competing interests

The authors declare that they have no competing interests.

## Authors’ contributions

YC carried out the literature review, study design, statistical analysis and drafted the manuscript. LC carried out the statistical analysis and edited the draft. TH helped with the literature review and statistical analysis. WK helped with study design and editing the draft. TC participated in the study design and coordination. YN helped with drafting the manuscript. SC participated in the study design. All authors read and approved the final manuscript.

## Pre-publication history

The pre-publication history for this paper can be accessed here:

http://www.biomedcentral.com/1472-6939/15/21/prepub
